# Assessing the expansion of the Cambrian Agronomic Revolution into fan-delta environments

**DOI:** 10.1038/s41598-022-18199-4

**Published:** 2022-08-24

**Authors:** Andrei Ichaso, Luis A. Buatois, M. Gabriela Mángano, Patty Thomas, Don Marion

**Affiliations:** 1 Department of Geological Sciences, University of Saskatchewan, 114 Science Place, Saskatoon, SK S7N 5E2 Canada; 2 North American Helium Inc., Suite 560, 440-2 Ave. S.W., Calgary, AB T2P 5E9 Canada

**Keywords:** Palaeoecology, Solid Earth sciences

## Abstract

The intensity, extent, and ecosystem-level impact of bioturbation (i.e. Agronomic Revolution) at the dawn of the Phanerozoic is a hotly debated issue. Middle Cambrian fan-delta deposits in southwestern Saskatchewan provide insights into the paleoenvironmental extent of the Agronomic Revolution into marginal-marine environments. The studied deposits reveal that several environmental stressors had direct impact on trace-fossil distribution and bioturbation intensities in Cambrian fan deltas. Basal and proximal subaerial deposits are characterized by very coarse grain size and absence of bioturbation. Mid-fan and fan-toe deposits were formed under subaqueous conditions and are characterized by rapid bioturbation events in between sedimentation episodes when environmental stressors were ameliorated, providing evidence of a significant landward expansion of the Agronomic Revolution. Transgressive marine deposits accumulated after the abandonment of the fan-delta system display high levels of bioturbation intensity, reflecting stable environmental conditions that favored endobenthic colonization. The presence of intense bioturbation in both subaqueous fan delta and transgressive deposits provides further support to the view that Cambrian levels of biogenic mixing were high, provided that stable environmental conditions were reached. Our study underscores the importance of evaluating sedimentary facies changes to assess the impact of environmental factors prior to making evolutionary inferences.

## Introduction

The Ediacaran-Cambrian represents a time of profound changes in terms of evolutionary innovations and geochemical cycles. Recent research has highlighted the geobiologic role of biogenic mixing by the rising bilaterian endobenthos in terms of its impact on ecosystem engineering, sediment rheology, and geochemical cycles^1–13^. However, the intensity and extent of bioturbation during the early Paleozoic and the impact that endobenthic colonization may have had at ecosystem scale is still a contentious topic^4^. A central topic in this debate is on the nature and significance of the Agronomic Revolution, which refers to the replacement of microbial matgrounds by biogenically reworked mixgrounds at the dawn of the Phanerozoic^4,14,15^. In this scenario, a wide variety of Ediacaran-style modes of life directly related to the exploitation of food resources contained within the microbial mat became increasingly restricted, with modern styles of animal-sediment interactions and feeding strategies becoming dominant^16^. There is growing evidence that this change was diachronic, starting during Cambrian Age 2 in shallow-marine settings and expanding progressively seaward into deep-sea settings and landward into marginal-marine environments through the early Paleozoic^3,17–22^. The details of the landward expansion remain unclear due to a paucity of studies dealing with animal-substrate interactions in brackish-water, marginal-marine environments^23,24^. Fan deltas are formed due to accumulation of sediments derived from an alluvial fan system and deposited into a standing body of water, mainly or entirely subaqueously^25,26^. Fan-delta deposits have been recognized in both the ancient record and the modern^27,28^. Although they occur in a variety of geologic settings, fan deltas are often associated with steep slopes and active tectonism, where sediment-gravity flow or alluvial processes dominate^29,30^. Fan deltas represent coarse-grained marine littoral systems dominated by hyperpycnal conditions^31^. Due to a combination of various stressors, such as fluvial discharge, high sedimentation rates and strong erosion, fan deltas represent harsh environments for benthic colonization^32–35^.

**Figure 1:**
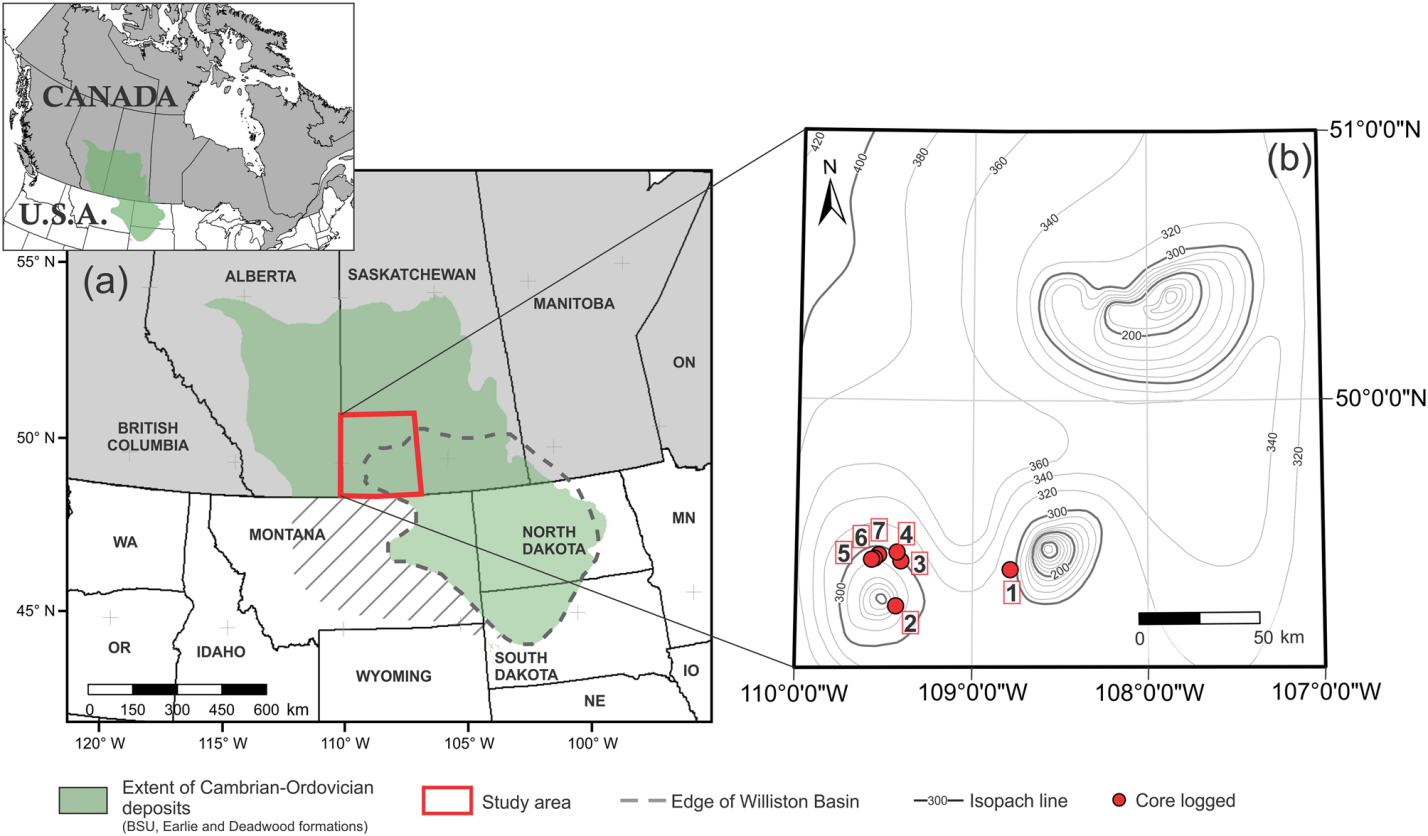
Location maps. (**a**) General map of western Canada and northwestern United States showing the approximate extent of Cambrian-Ordovician deposits in subsurface and the study area in Saskatchewan. Map generated by A.I. using ESRI ArcMap v. 10.5.0.6491. (**b**) Close-up of the study area showing well locations of the cores analyzed in this paper (SI Appendix, Table S1). Isopach map generated in ESRI ArcMap v. 10.5.0.6491 using data from^36^ and modified by A.I. in CorelDRAW 2017 v.19.1.0.419 software.

Cambrian cored intervals recovered at the flanks of inferred active blocks in southwestern Saskatchewan (Fig. 1) record a transition from terrestrial to marine settings. Cambrian fan-delta coarse-grained deposits rest atop Precambrian basement topographic highs, in places interfingering with tide-influenced, fine-grained deposits. These deposits offer a unique opportunity to gain insight into the depositional processes and endobenthic colonization history of Cambrian fan-delta systems, providing a glimpse into the role of biogenic mixing in highly stressful settings at the dawn of the Phanerozoic. Therefore, the objectives of this study are to: (1) integrate sedimentologic and ichnologic datasets to reconstruct animal-substrate interactions in this Cambrian fan-delta system, and (2) assess the implications of our analysis within the framework of the landward expansion of the Agronomic Revolution as revealed by intense biogenic reworking.

## Geologic setting

Cambrian-Ordovician deposits are present in Saskatchewan subsurface throughout the southern half of the province. These deposits were accumulated within a shallow embayment formed by the flooding of the North American craton during an eastward expanding transgressive event. This embayed coastline extended south into the United States. It was limited to the north by the Peace River Arch, to the east by the Canadian Shield, and at the south end by the Transcontinental Arch (SI Appendix, Fig. S1). This embayment transitioned into more open shallow-marine environments later in the early Paleozoic. The dominantly clastic Paleozoic succession is commonly divided into three units: the middle Cambrian Basal Sandstone Unit and Earlie Formation, and the upper Cambrian to Lower Ordovician Deadwood Formation (SI Appendix, Fig. S2). These units regionally thicken to the western side of the province, reaching a maximum thickness of approximately 500 m at the Lloydminster depocenter, and thinning to the north and to the east. Localized thinning of the Cambrian deposits occurs in southwestern Saskatchewan, in areas where the Precambrian paleorelief was apparently irregular and contained numerous positive features, roughly southwest-northeast oriented^36,37^. Some of these structural highs have been interpreted as monadnocks, isolated hills that were temporarily exposed during Cambrian times and acted as detrital sources until drowned by the transgressive sea^38,39^. Other major structural blocks in the area, such as the Swift Current Platform, are considered to have influenced sedimentation across the entire Phanerozoic^40,41^.

The studied deposits correspond to the Basal Sandstone Unit and Earlie Formation and accumulated adjacent to these positive elements. Previous studies of these units in the target area are limited as the number of deep wells that penetrate Cambrian-Ordovician rocks were quite low and core availability is sparse compared to other parts of the province. Analyzed cores revealed unique coarse-grained facies that have not been reported in these units within the Western Canada Sedimentary Basin before.

## Results

These deposits were divided into fifteen facies and grouped into two broad facies associations: fan-delta and open-bay deposits (SI Appendix, Tables S2 and S3). Fan-delta deposits were characterized following conventional terminology^25,26^. Subdivisions used for open-bay deposits follow previously proposed schemes for this environment^33,42^.

### Facies association 1 (FA1): proximal to distal fan-delta deposits

#### Description

**Figure 2:**
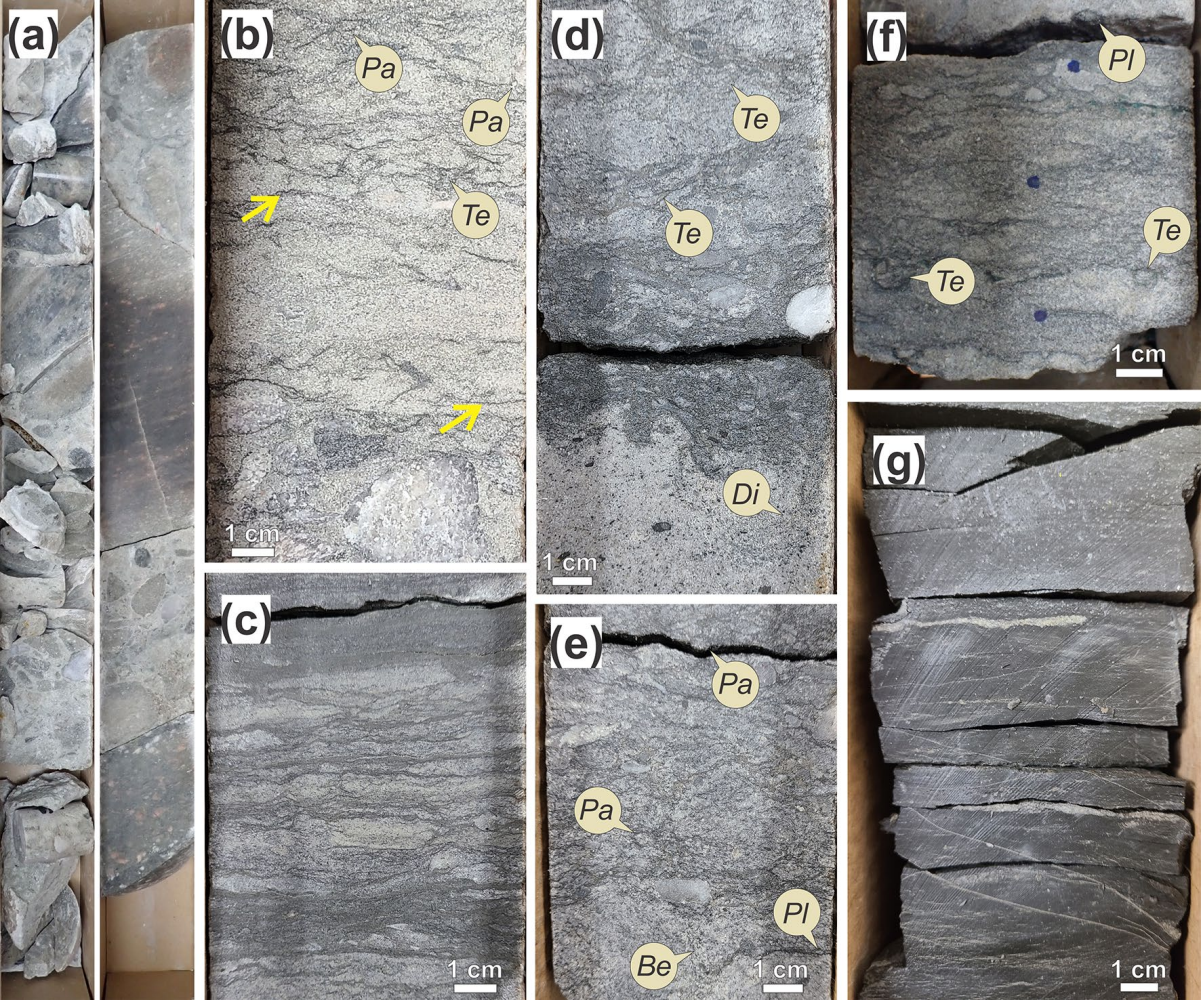
Trace fossils and sedimentary facies of Facies Association 1 (FA1). (**a**) General view of granule to boulder conglomerate (Cg). Core width 7.5 cm. (**b**) *Teichichnus rectus* (*Te*) and *Palaeophycus* isp. (*Pa*) in interbedded sandstone and conglomerate (S-Cg). Sandstone bed is mudstone draped (yellow arrows) forming flaser and wavy bedding. (**c**) Argillaceous sandstone and mudstone (Sd). (**d**) *Teichichnus rectus* (*Te*) and poorly defined *Diplocraterion* isp. (*Di*) penetrating in bioturbated gravelly sandstone (Sg-bio). (**e**) *Palaeophycus* isp. (*Pa*) and *Planolites* isp. (*Pl*) in bioturbated gravelly sandstone (Sg-Bio). *Bergaueria* isp. (*Be*) preserved at base of sandstone layer. (**f**) *Teichichnus rectus* (*Te*) and *Planolites* isp*.* (*Pl*) in argillaceous sandstone and mudstone (Sd). (**g**) Parallel-laminated shale (Sh). Photographs taken by A.I. Labels created by A.I. using CorelDRAW 2017 v.19.1.0.419 software.

This facies association is dominated by very coarse- to medium-grained sandstone and granule to boulder conglomerate. It constitutes the Basal Sandstone Unit at the flanks of Precambrian highs. This facies association forms 3–22 m thick upward-fining intervals and tends to be abruptly overlain by FA2. FA1 is composed of facies Cgb, S-Cg, Sg, Sg-bio, Sd, and locally facies Sh (Fig. 2). Granule to boulder conglomerate (Cgb) (Fig. 2a), generally ungraded, is commonly found at the base of the succession, forming dm- to m- thick beds, and resting on top of metamorphic and igneous Precambrian rocks. Basal conglomerate beds are gradationally overlain by interbedded sandstone and pebble conglomerate (S-Cg) (Fig. 2b) with rare mudstone drapes. Locally, basal conglomerate beds are sharply overlain by massive gravelly sandstone (Sg) (Fig. 2d). Bioturbated sandstone (Sg-bio) (Fig. 2e) and argillaceous sandstone (Sd) (Fig. 2c,f) are particularly common in the upper intervals of FA1. Parallel-laminated shale (Fig. 2g) is locally observed forming intervals up to one meter thick.

#### Trace and body fossils

Bioturbation degree in FA1 is variable, ranging from unbioturbated to highly bioturbated (BI 0–4) deposits. Bioturbation intensity increases upwards in the succession. Trace-fossil diversity is low. Basal conglomerate layers are barren. Common trace fossils in mudstone-draped intervals include *Teichichnus rectus* (Fig. 2b,d,f), *Planolites* isp. (Fig. 2e,f)*, Palaeophycus* isp. (Fig. 2b,e)*,* and *Bergaueria* isp. (Fig. 2e). A single occurrence of *Diplocraterion* isp. (Fig. 2d) is observed in gravelly sandstone. Parallel-laminated shale is unbioturbated. FA1 upper intervals are fossiliferous. Shells of linguliform brachiopods are commonly found in parallel-laminated mudstone and mudstone-draped fine-grained sandstone, in places aligned to internal cross laminations.

#### Interpretation

Ungraded boulder conglomerate represents individual erosive and non-cohesive debris-flow deposits accumulated at the top of a fan delta with prevalent subaerial conditions. Sg represents a transition between the subaerial and subaqueous sections of the fan delta, evidenced by rare vertical burrows (*Diplocraterion* isp.) at bed tops. Facies S-Cg and Sg-bio are genetically related and represent the mid-fan delta. These facies were deposited in subaqueous conditions with sediments probably being reworked by weak tidal currents as suggested by the pervasive presence of mudstone drapes forming flaser and wavy bedding. Facies Sd records deposition at the toe of the fan and its finer-grained sandstone evidences waning flow conditions. Facies SH records suspension-fallout deposition in low-energy sheltered areas, probably formed between fan wedges.

The low ichnodiversity and sparse bioturbation in FA1 indicate stressful conditions for benthic colonization. In particular, the lack of bioturbation in basal conglomerate and gravelly sandstone probably is due to a combination of subaerial conditions, high energy, and sedimentation rate. In turn, absence of bioturbation in local parallel-laminated mudstone intervals suggests stressful brackish-water conditions in a low-energy, protected area. The presence of linguliform brachiopods in these deposits is also consistent with brackish-water conditions. Deposit-feeding structures (*Teichichnus rectus*, *Planolites* isp.) dominate the assemblage, suggesting that organic particles were more abundant in the sediment. Horizontal structures of deposit feeders are common in mudstone-draped intervals, recording higher burrowing activity during periods of low energy and suspension fallout. Although rare, the local presence of dwelling traces of suspension-feeders (*Palaeophycus* isp.*, Diplocraterion* isp.) suggests limited feeding from suspended particles. Passive predation is indicated by *Bergaueria* isp., typically attributed to sea anemones^43^.

### Facies association 2 (FA2): open-bay deposits

#### Description

**Figure 3:**
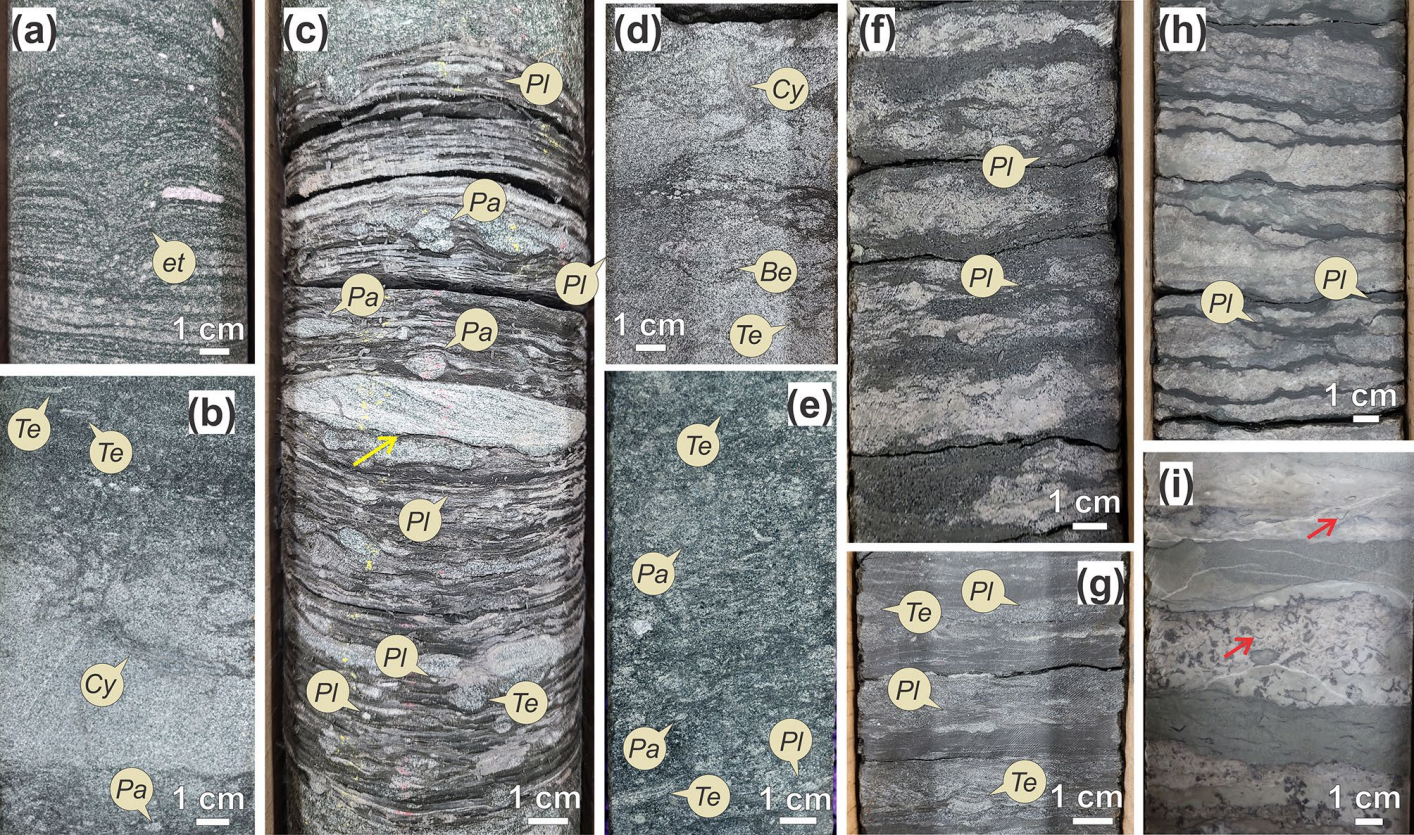
Trace fossils and sedimentary facies of Facies Association 2 (FA2). (**a**) Escape trace fossil (et) in cross-bedded glauconitic sandstone (Sxgl). (**b**) *Teichichnus rectus* and inclined *Cylindrichnus concentricus* in interbedded glauconitic sandstone and mudstone (Sgl-Mgl). (**c**) Parallel-laminated green mudstone (Mg) with very thin-bedded glauconitic sandstone layers and cross-laminated sand lenses (yellow arrow). *Teichichnus rectus* (*Te*), *Palaeophycus* isp. (*Pa*), and *Planolites* isp. (*Pl*). (**d**) Small *Cylindrichnus concentricus* and *Bergaueria* isp. (*Be*) in glauconitic sandstone (Sxgl). Scale bar is 1 cm. (**e**) Moderate to highly bioturbated glauconitic sandstone (Sgl-bio). *Teichichnus rectus* (*Te*)*, Palaeophycus* isp. (*Pa*), and *Planolites* isp. (*Pl*). (**f**) Interbedded glauconitic sandstone and limestone (Sgl-Lst) with *Planolites* isp. (*Pl*). (**g**) *Teichichnus rectus* (*Te*) and *Planolites* isp. (*Pl*) in interbedded glauconitic sandstone and mudstone (Sgl-Mgl). Note the presence of discrete thin intervals with undetermined bioturbation mottling. (**h**) Interbedded mudstone and limestone (M-Lst). (**i**) Limestone with discontinuous mudstone and trilobite and linguliform brachiopod fragments (red arrows). Photographs taken by A.I. Labels created by A.I. using CorelDRAW 2017 v.19.1.0.419 software.

FA2 consists of various glauconite-rich, coarse- to very fine-grained sandstone and mudstone facies with interbedded carbonate rocks. This facies association is part of the Earlie Formation. FA2 abruptly overlies FA1 and commonly forms an overall fining- and deepening-upward succession. Cored FA2 intervals are 12–45 m thick; however, gamma ray responses suggest FA2 deposits could be even thicker (up to 65 m thick). This facies association encompasses facies Sgl-Cgl, Sxgl, Sgl-Mgl, Mg, Sgl-Bio, Sgl-Lst, M, M-Lst, and Lst (Fig. 3). Glauconitic sandstone and conglomerate (Sgl-Cgl) layers commonly rest at the contact between FA1 and FA2, as this facies erosively overlies facies Sg and Sd. Lower intervals of FA2 are dominated by sandstone-rich and glauconitic facies. Facies Sgl-Cgl, Sxgl, Sgl-Mgl, Mg, and Sgl-Bio are genetically related, and locally form coarsening-upwards cycles. Parallel-laminated green mudstone intercalated with discontinuous, fine- to very fine-grained sandstone (Mg) (Fig. 3c) is commonly overlain by interbedded wave-ripple cross-laminated, fine- to very fine-grained sandstone and mudstone (Sgl-Mgl) (Fig. 3b,g), and by bioturbated, glauconitic, fine-grained sandstone (Sgl-bio) (Fig. 3e). The coarsening-upward cycles are capped by sharp-based, trough cross-bedded, glauconitic, fine- to medium-grained sandstone (Sxgl) (Fig. 3a,d) and gravelly sandstone (Sgl-Cgl). The upper intervals of FA2 are carbonate- and shale-dominated. Interbedded green, fine-grained sandstone and dolostone (Sgl-Lst) (Fig. 3f) commonly represent the transition between the lower glauconite-rich interval and the less glauconitic upper interval of FA2. Parallel-laminated mudstone (M) with discontinuous carbonate layers and nodules form uniform successions, several meters thick (3–15 m). Carbonates in facies Lst (Fig. 3i) and M-Lst (Fig. 3h) are commonly nodular and argillaceous; however, carbonate clasts are locally present. There is an exception to the general FA2 facies distribution in wells 101/03-35-005-27W3/0 and 02/11-23-005-26W3/0, where glauconitic facies are missing and basal conglomerates (Cg) of FA1 are sharply overlain by mudstone and carbonate beds instead (M-Lst). These wells are in a more proximal position to the crest of the uplifted blocks.

#### Trace and body fossils

Bioturbation degree in FA2 ranges from absent to very high (BI 0–5). Bioturbation intensity is higher (BI 2–5) in glauconitic, fine-grained sandstone with dominance of feeding structures made by deposit feeders (Fig. 3e). Thin intervals characterized by indistinct bioturbation mottling occur in these deposits. The trace-fossil assemblage is dominated by *Planolites* isp. (Fig. 3c,f,g) and *Teichichnus rectus* (Fig. 3b,e,g) with subordinate *Bergaueria* isp. (Fig. 3d), *Cylindrichnus concentricus* (Fig. 3b,d)*, Palaeophycus* isp. (Fig. 3c), and *Skolithos linearis*. Local escape trace fossils (Fig. 3a) occur in cross-bedded glauconitic sandstone (Sxgl). Parallel-laminated mudstone and interbedded mudstone and carbonate intervals are unbioturbated to sparsely bioturbated (BI = 0–2), hosting a few *Planolites* isp. (Fig. 3h), *Helminthopsis* isp., and unidentified small trails on bedding surfaces. Fine-grained facies of FA2 are fossiliferous, containing abundant fragments of linguliform brachiopods, hyoliths, and trilobites. Some limestone beds are also fossiliferous. Preliminary trilobite identification suggests the specimens collected belong to *Kootenia quadriceps*^44^, *Modocia* sp.^45^, *Asaphiscus wheeleri*^46^, and *Blainia gregaria*^47^, both middle Cambrian in age (N. Handkamer, personal communication, January 10, 2022).

#### Interpretation

FA2 represents proximal to distal embayment deposits. Facies Sgl-Cgl on top of FA1 represent transgressive lag deposits and the abandonment of the fan-delta system. Facies Mg records deposition by suspension fallout in a low-energy setting between storm wave base and fair-weather wave base. Parallel-laminated sandstone in facies Mg represents distal tempestites. Facies Sgl-Mgl and Sgl-Bio record deposition just below fair-weather wave base, in the distal bay zone, reflecting frequent alternation of quiet water sediment fallout interrupted by combined-flow deposition during storm events. Facies Sgl-Cgl and Sxgl record deposition under moderate- to high-energy conditions, above fair-weather wave base, representing proximal bay deposits. Facies M, Sh, M-Lst, and Lst record mainly deposition below the storm wave base with dominance of suspension fallout, representing shelf settings. Facies M-Lst and Lst fossiliferous limestone and dolostone are considered to have formed by precipitation of calcium carbonate from seawater and post-depositional dolomitization. Local carbonate clasts in mudstone-dominated facies indicate erosion, transportation, and deposition of calcareous material from a nearby carbonate platform. Facies Sgl-Lst is found in well 101/02-27-005-27W3 on top of glaucony facies. It represents a mixed setting with important clastic supply and carbonate precipitation that transitions into a shelf.

Compared to FA1, FA2 deposits show higher bioturbation intensities and a slightly higher ichnodiversity. The dominance of feeding and dwelling structures of deposit and detritus feeders (*Planolites* isp., *Teichichnus rectus, Cylindrichnus concentricus*) indicates a low-energy and organic-rich environment. Escape trace fossils and dwelling structures of suspension-feeders (*Skolithos linearis*, *Palaeophycus* isp.) in glauconitic coarse-grained sandstones indicate a shifting sandy environment of moderate- to high-energy in which waves and currents kept organic particles in suspension. The presence of linguliform brachiopods, hyoliths, and trilobites is consistent with deposition in an open-bay setting.

## Discussion

Cored intervals sampled at the flanks of Precambrian highs reveal a succession that at its base is dominated by deposits representing the subaerial portion of fan deltas. No deposition is envisaged in the study area during forced regression (i.e. falling stage systems tract). The first stage of sedimentation is represented by lowstand systems tract deposits (SI Appendix, Figs. S4, S5 and S6A). Initial base-level rise over the craton in southwestern Saskatchewan resulted in the creation of accommodation space, rapidly filled by prograding to aggrading fan deltas during normal regression. This lowstand stage is recorded in the succession by stacked conglomeratic beds of the inner fan, forming bedsets up to 16 m thick.

Subsequent transgression led to landward migration of the fan-delta system and its final abandonment once completely flooded during the middle Cambrian. With continuous transgression, the rate of accommodation eventually exceeded the rate of sedimentation causing the shift of the shoreline in a landward direction; this is reflected in the gradual upwards deepening of fan-delta facies. The onset of the transgressive systems tract is represented by sandy, bioturbated, subaqueous mid and toe fan deposits gradually overlying unbioturbated subaerial proximal fan deposits, forming an overall fining-upward succession (SI Appendix, Figs. S4 and S5). Sustained sea-level rise led to the establishment of an embayment depositional setting, and proximal to distal bay deposits accumulated (SI Appendix, Fig. S6B). As transgression continued and elevated areas were flooded, the rate of sediment supply was greatly diminished, and both storm and fair-weather waves reworked the outer delta deposits, forming sandy proximal embayment complexes. Wave action is revealed by the presence of symmetrical and combined-flow ripples. A decrease in the salinity stress and a transition to near-normal marine conditions are evidenced by more continuous bioturbation, abundant body fossils, and the formation of glauconite in areas of low energy and low sedimentation rates. Once formed, glauconite grains were transported and accumulated by storm and wave currents producing a distinctive green facies^48^. Glauconitic sandstone commonly lies sharply on top of outer fan-delta facies and overlain in turn by distal bay to shelf facies. Locally, transgressive lag deposits separate proximal bay from fan-delta facies, indicating pronounced transgressive erosion. As the transgression continued, the shoreline migrated landwards, and a deepening of facies occurred. Well cores in a more distal position (101/11-23-005-26W3/00) from the source area show shelf mudstone erosively overlying proximal fan-delta facies. In these areas, mid and toe fan facies were completely removed due to wave ravinement as the sea transgressed. Thick mudstone accumulated in the shelf area, below the storm wave base (SI Appendix, Fig. S6B,C). Warm and relatively shallow-water conditions were favorable for carbonate precipitation, as indicated by the presence of limestone and dolostone.

**Figure 4:**
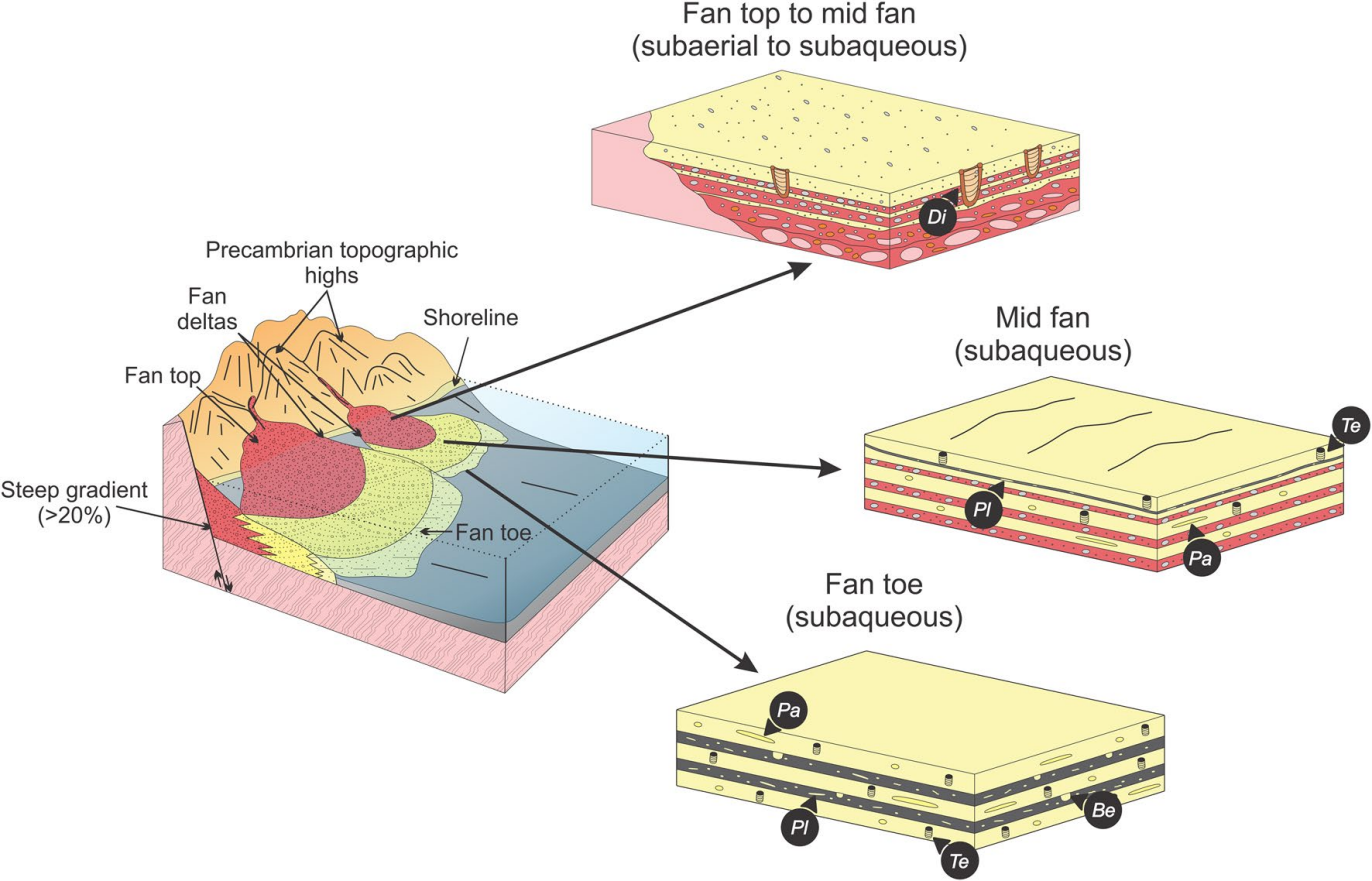
Block diagram showing trace-fossil distribution in Cambrian fan-delta subenvironments. Block diagrams drawn by A.I. using CorelDRAW 2017 v.19.1.0.419 software.

Fan-delta deposits in southwestern Saskatchewan are gravelly and characterized by thick conglomerate containing boulder-size clasts, with no apparent grading, that transition downflow into conglomeratic sandstone and mudstone-draped sandstone (SI Appendix, Fig. S3). These deposits are associated with steep and tectonically active scarps, and commonly developed a conical- to wedge-shape geometry^25^. Tidal amplification could have been promoted by the development of irregular and protected semi-enclosed areas at the front of and between fan wedges (SI Appendix, Fig. S6A). In subsurface Saskatchewan, trace fossils were key to differentiate between the subaerial and subaqueous segments of fan deltas. The Cambrian interval records the initial forays of benthic organisms into shallow- and marginal-marine settings. Colonization of continental settings by animals did not occur until Silurian times, therefore bioturbation in fluvial and subaerial settings was most likely excluded^23,49–51^. In contrast, subaqueous fan-delta deposits in southwestern Saskatchewan are characterized by the presence of bioturbation structures, recording short-term to even more continuous colonization windows for the burrowing endobenthos (Fig. 4).

**Figure 5:**
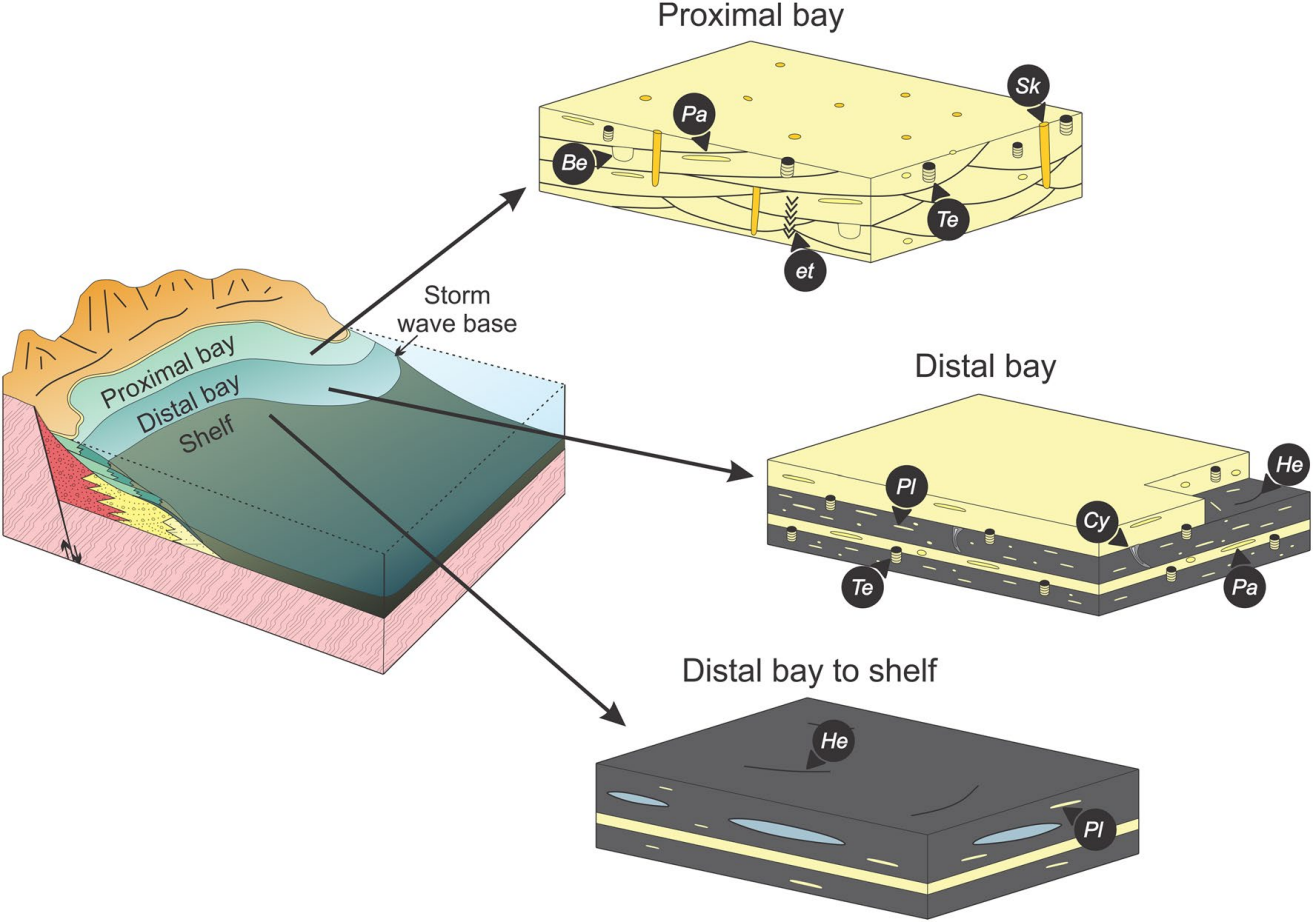
Block diagram showing trace-fossil distribution in the Earlie Formation proximal bay to shelf subenvironments. Block diagrams drawn by A.I. using CorelDRAW 2017 v.19.1.0.419 software.

Cambrian alluvial plains were essentially devoid of plant cover and typified by poorly constrained, low-sinuosity sheet-braided fluvial systems^52^. The absence of vegetation cover was detrimental for fine-grained sediment retention and alluvial plains were mud-deficient^53^. This configuration of the alluvial landscape promoted episodic sediment delivery to coastal and shelfal areas which was mostly controlled by discharge variations in fluvial catchment areas^52^. Cambrian fan-delta strata in Saskatchewan present some common points with pre-vegetation settings described in previous works^54–58^, such as very thick (1–3 m) sand- to gravel-rich deposits, overall low mud content, and abundant sandy bedforms. Subaqueous mid-fan coarse-grained deposits are characterized by low to absent bioturbation, reflecting stressful conditions resulting from a combination of high-energy, high sedimentation rates, and possible salinity dilution during episodes of increased fluvial discharge. It was only during brief periods of quiescence that endobenthic activity took place, evidenced by the presence of sparse trace fossils associated with mudstone drapes, reflecting short-term colonization windows. In contrast to the more stressful conditions of the mid fan delta, argillaceous and tide-influenced outer fan facies display an increase in bioturbation intensity, indicating a more continuous colonization window. Despite an increase in the intensity of bioturbation in fan-toe deposits, ichnodiversity remained relatively low, suggesting that reduced salinity was a significant stressor. Transgressive glauconitic deposits overlying fan-delta strata show higher levels of endobenthic activity, higher ichnodiversity, and common marine body fossils, reflecting an increase in salinity and less stressful conditions (Fig. 5).

The studied deposits allow assessing the paleoenvironmental extent of the Agronomic Revolution in marginal-marine settings. In particular, the impact of bioturbation during the early Paleozoic has been a topic of debate with contrasting views regarding the intensity of biogenic reworking in Cambrian deposits (see discussion in^4^). The implications of our study are four-fold. First, integration of ichnologic and sedimentologic datasets within a stratigraphic framework suggests that bioturbation intensity reached high levels under stable environmental conditions. This is illustrated by the fact that transgressive deposits representing accumulation in open-bay and shelf environments, formed under near-normal marine salinity conditions, low energy, and well-oxygenated conditions, tend to be intensely bioturbated. Second, even short-term periods of amelioration in environmental stressors allowed for moderate intensities of biogenic reworking to occur. These rapid bioturbation events are clearly displayed in the fan-delta toe deposits, recording endobenthic colonization during times in between sedimentation episodes. These two points taken together support the view that Cambrian levels of bioturbation intensity were high provided stable environmental conditions were met. Third, the occurrence of trace fossils in both mid fan delta and fan-delta toe deposits provides evidence of significant landward expansion of the Agronomic Revolution. This is consistent with several studies that have documented trace fossils in a wide variety of Cambrian marginal-marine environments, such as bays, deltas, and estuaries^23,24,56,59–64^. We note that fan deltas, given their coarse grain size, rapid deposition, and very high hydrodynamic energy, may be regarded as representing an end member in terms of stressor intensities if compared with other marginal-marine environments, such as interdistributary bays or estuarine basins. Fourth, our study underscores the importance of carefully evaluating subtle changes in the nature of sedimentary facies (e.g. heterolithic deposits) in order to assess the impact of environmental factor prior to making inferences in evolutionary terms.

Many studies of fan deltas focus on the sedimentary facies, depositional processes, and geometry of such systems, but only a few integrate sedimentologic and ichnologic observations^32,34,65,66^. Notably, most of these studies deal with post-Paleozoic fan deltas, being the study by^66^ on a Pennsylvanian system a notable exception. Expanding the fan-delta ichnology dataset would be essential to track secular changes in style of bioturbation in these environments through geologic time. Ideally, this would require documentation of the trace-fossil content of the various fan-delta subenvironments and a large dataset including systems of different ages and geologic contexts.

## Materials and methods

This study is based on the analysis of seven continuous well cores (SI Appendix, Table S1) drilled at the flanks of basement highs in southwestern Saskatchewan. Cored intervals were logged in detail and the deposits were divided into sedimentary facies based on lithology, bed contacts, bed thickness, color, grain size, mineralogy, physical sedimentary structures, and trace-fossil content and distribution. The facies were grouped into two facies associations.

Classification of sandstone was based on generally accepted schemes^67^. The classic Udden-Wentworth scale^68^ was used for classifying grain-size and visual estimations of roundness and sorting followed current practices^69^. Bed thickness was classified after previous terminology^70^. Glauconite content was visually estimated by using percent abundance charts.

Ichnologic data comprise ichnotaxon identification to ichnospecies where possible, trophic types, degree of bioturbation, and cross-cutting relationships. Degree of bioturbation was estimated using the system proposed by Taylor and Goldring^71^, and a bioturbation index grade (BI), ranging from 0 (undisturbed bed) to 6 (complete bioturbation and sediment reworking) was provided.

## Supplementary Information

Supplementary Information.

## Data Availability

The data that support the findings of this study are available from the corresponding author, A.I., upon reasonable request.
