# Supplementary material for: Assessing the expansion of the Cambrian Agronomic Revolution into fan-delta environments
Source: Sci Rep. 2022 Aug 24;12:14431. doi: 10.1038/s41598-022-18199-4 (PMC9402710; doi:10.1038/s41598-022-18199-4)
Supplement: Supplementary file 1 — Supplementary Information. [file 41598_2022_18199_MOESM1_ESM.pdf]

Supplementary Materials for

Assessing the expansion of the Cambrian Agronomic Revolution into fan-delta environments

Andrei Ichaso[1,*], **Luis A. Buatois**[1], **M. Gabriela Mángano**[1], **Patty Thomas**[2] **and Don Marion**[2]

[1] Department of Geological Sciences, University of Saskatchewan, 114 Science Place, Saskatoon, Saskatchewan, S7N 5E2, Canada.

[2] North American Helium Inc. Suite 560, 440-2 Ave. S.W., Calgary, Alberta, T2P 5E9, Canada.

[*] Corresponding author

This PDF file includes:

Tables S1-S3

Figures S1–S4

References

List of supplementary materials:

Table S1. List of cores logged in southwestern Saskatchewan

Table S2. Fan-delta sedimentary facies of the middle Cambrian in southwestern Saskatchewan

Table S3. Open-bay and transgressive sedimentary facies of the middle Cambrian in southwestern Saskatchewan

Figure S1. Paleogeographic map of the late Cambrian (ca. 500 Ma)

Figure S2. Cambrian-Silurian stratigraphy in Alberta and Saskatchewan

Figure S3. Architecture of graven-rich fan deltas

Figure S4. SW-NE stratigraphic correlation in the Cypress West area

Figure S5. NW-SE stratigraphic correlation in the Cypress East area

Figure S6. Paleoenvironmental evolution of successions close to uplifted and active Precambrian blocks

Table S1. List of cores logged in southwestern Saskatchewan.

No. on Fig. 1	Well ID	License Number	Core Interval(s)
1	101/12(6)-09-005-21W3	128157	2525.00-2559.00 m
2	101/04-27-003-26W3	165696	2386.00-2406.90 m
3	101/11-23-005-26W3	134457	2412.00-2433.90 m
4	101/14-34-005-26W3	126469	2424.00-2437.00 m
5	101/16-21-005-27W3	164291	2403.00-2453.43 m
6	101/02-27-005-27W3	164293	2448.00-2511.00 m
7	101/03-35-005-27W3	162932	2399.00-2435.00 m

Table S2. Fan-delta sedimentary facies of the middle Cambrian in southwestern Saskatchewan

FACIES	LITHOLOGY AND SEDIMENTARY STRUCTURES	DEPOSITIONAL PROCESSES	BIOTURBATION DEGREE AND TRACE FOSSILS	PALEOENVIRONMENTAL DISTRIBUTION	LITHOSTRATIGRAPHIC UNIT
Cgb	**Granule to boulder conglomerate):** Facies interval: 3-16 m thick. Tan to greenish gray, very thick- to thick-bedded (>100 cm thick), erosive-based, very poorly to moderately sorted, structureless to graded, matrix- to grain-supported, polymictic, granule to boulder conglomerate. Matrix is composed of sub-angular to sub-rounded, coarse sand-size quartz grains with accessory pyrite and muscovite. Clasts are sub-angular to sub-rounded, sub-horizontally to sub-vertically oriented, 1 cm to >200 cm in size, and are predominantly quartz and basement rocks.	Very high to high energy. Sediment gravity flow deposits. Lack of abundant fines and inconsistent grading suggest non-cohesive debris flows. Absence of bioturbation supports subaerial deposition.	BI = 0	Fan delta: fan top, subaerial	Basal Sandstone Unit
Sg	**Gravelly sandstone** Facies interval: 0.1-0.7 m thick. Gray, medium-bedded (10-30 cm thick), sharp-based, massive to horizontally bedded, medium- to very coarse-grained sandstone. Common quartz and lithic granules and pebbles. Local trace fossils at the top of beds.	Very high to high energy. Dominant bed-load deposition. Occurrence of the ichnogenus *Diplocraterion* indicates that some intervals were formed under marine influence.	BI = 0-1<br><br>*Diplocraterion* isp.	Fan delta: fan top to mid-fan, subaerial to subaqueous	Basal Sandstone Unit
S-Cg	**Interbedded sandstone and conglomerate** Facies interval: 1-3 m thick. Intercalated gray to tan, very thin- to medium-bedded (2-23 cm thick), sharp-based, faintly cross-bedded, fine- to coarse-grained quartz sandstone with local mudstone drapes, and gray to green, thin- to medium-bedded (4-47 cm thick), sharp- to erosive-based, very poorly- to moderately-sorted, polymictic, matrix-supported pebble conglomerate. Common fining-upward trends. Mudstone drapes become more abundant in fine-grained sandstone layers and form wavy and flaser bedding. Sparse to moderate intensity of bioturbation in mudstone-draped fine-grained sandstone layers, conglomerate and coarse- to medium-grained sandstone are barren. Local linguliform brachiopod shell fragments.	Very high to high energy. Bed-load deposition. Mudstone drapes reflect alternation of bed-load deposition and suspension fallout. Ichnologic evidence indicates marine deposition.	BI = 0-3<br><br>*Planolites* isp.<br>*Palaeophycus* isp.<br>*Teichichnus rectus*	Fan delta: mid-fan, subaqueous	Basal Sandstone Unit
Sg-Bio	**Bioturbated gravelly sandstone** Facies interval: 0.4-5 m thick. Amalgamated gray to tan, medium-bedded (11-20 cm thick), flaser- to wavy-laminated, horizontally- to planar cross-bedded, poor to well-sorted, coarse-grained to gravelly sandstone with discontinuous mudstone drapes. Normal gradation. Common granules and pebbles. Mudstone laminae between beds.	High energy. Bed-load deposition with suspension fallout during slack water periods. Ichnologic evidence indicates marine deposition.	BI = 1-3<br><br>*Teichichnus rectus*<br>*Planolites* isp.<br>*Palaeophycus* isp<br>*Bergaueria* isp.	Fan delta: mid-fan, subaqueous	Basal Sandstone Unit
Sd	**Argillaceous sandstone and mudstone** Facies interval: 1.5-2.5 m thick. Interbedded gray, thin- to medium-bedded (4-11 cm thick), sharp-based, ripple cross-bedded, fine- to medium-grained sandstone with mudstone drapes and dark gray, very thin- to thin-bedded (<1 to 4 cm thick), wavy laminated mudstone. Local gray, 10-30 cm thick, cross-bedded, medium- to coarse-grained sandstone. Abundant linguliform brachiopod shell fragments aligned with cross-bedding.	Low to moderate energy. Dominant bedload deposition alternating with suspension fallout during periods of low flow velocities. Deposition under tidal influence. Bioturbation associated to slack water periods.	BI = 1-4<br><br>*Teichichnus rectus*<br>*Planolites* isp.<br>*Palaeophycus* isp.<br>*Bergaueria* isp.	Fan delta: toe (outer fan), subaqueous	Basal Sandstone Unit

Table S2. (Continued)

FACIES	LITHOLOGY AND SEDIMENTARY STRUCTURES	DEPOSITIONAL PROCESSES	BIOTURBATION DEGREE AND TRACE FOSSILS	PALEOENVIRONMENTAL DISTRIBUTION	LITHOSTRATIGRAPHIC UNIT
Sh	**Parallel-laminated shale**<br>Facies interval: 0.3-1 m thick. Dark gray to black, medium- to thick-bedded (30-100 cm thick), parallel-laminated, non-calcareous shale with local siltstone laminae. Abundant linguliform brachiopod shell fragments.	Low energy. Suspension fall-out Deposition in a protected setting.	BI = 0	Protected areas between fan-delta wedges.	BSU

Table S3. Open-bay and transgressive sedimentary facies of the middle Cambrian in southwestern Saskatchewan

FACIES	LITHOLOGY AND SEDIMENTARY STRUCTURES	DEPOSITIONAL PROCESSES	BIOTURBATION DEGREE AND TRACE FOSSILS	PALEOENVIRONMENTAL DISTRIBUTION	LITHOSTRATIGRAPHIC UNIT
Sgl-Cgl	**Glauconitic coarse-grained and gravelly sandstone**<br>Facies interval: 0.1-0.3 m thick. Green, very thin- to thick-bedded (2 to 30 cm thick), sharp- and erosive-based, massive to cross-bedded, poorly- to moderately-sorted, medium- to coarse-grained to pebbly, glauconitic sandstone. Normally-graded foresets. Disseminated pyrite and nodules. Glauconite content highly varies (5-20%). Linguliform brachiopod shell fragments.	Moderate to high energy. Bed-load deposition. Pebbly sand layers represent storm events lag deposits. Bioturbation reflects common physical instability of the substrate. Marine setting.	BI = 0-1<br><br>*Planolites* isp.<br>*Skolithos linearis*<br>*Palaeophycus* isp.	Transgressive deposits: transgressive lag.	Earlie Formation
Sxgl	**Cross-bedded glauconitic sandstone with mudstone laminae**<br>Facies interval: 0.1-0.5 m thick. Gray to green, thin-bedded (10-45 cm thick), planar to trough cross-bedded, moderately- to well-sorted, fine- to medium-grained, glauconitic sandstone with rare dark gray to green, laminae to very thin-bedded (<1-3 cm thick), bioturbated mudstone. Local 2-3 cm wide lithic clasts. Glauconite content is 15 to 30%. Few linguliform brachiopod shell fragments.	High energy. Bed-load deposition. Migration of 2D and 3D dunes. Marine setting.	BI = 0-2<br><br>*Teichichnus rectus*<br>*Skolithos linearis*<br>*Bergaueria* isp.<br>*Planolites* isp.<br>Escape trace fossils	Transgressive deposits: proximal bay	Earlie Formation
Mg	**Parallel-laminated green mudstone**<br>Facies interval: 0.2-0.6 m thick. Dark green, thin- to medium-bedded (10-60 cm thick), parallel-laminated bioturbated mudstone with green, very thin-bedded (1-3 cm thick), cross-laminated, glauconitic, very fine- to fine-grained sandstone and sandstone lenses. Trilobite fossils and small linguliform brachiopod shell fragments.	Low energy. Dominated by suspension-fallout of fine-grained particles with occasional storm events. Deposition between fair-weather wave base and storm wave base.	BI = 0-2<br><br>*Planolites* isp.<br>*Teichichnus rectus*	Transgressive deposits: distal bay	Earlie Formation

Table S3. (Continued)

FACIES	LITHOLOGY AND SEDIMENTARY STRUCTURES	DEPOSITIONAL PROCESSES	BIOTURBATION DEGREE AND TRACE FOSSILS	PALEOENVIRONMENTAL DISTRIBUTION	LITHOSTRATIGRAPHIC UNIT
Sgl-Mgl	**Interbedded parallel-laminated glauconitic sandstone and mudstone**<br>Facies interval: 0.3-1.5 m thick. Regular interbedding of dark gray to greenish gray, laminae to very thin-bedded (<1-3 cm thick), wavy- to parallel-laminated, mudstone and green, laminae to very thin-bedded (<1-2 cm thick), sharp-based, parallel- to cross-laminated, glauconitic, very fine- to fine-grained sandstone. Linguliform brachiopod shell fragments on bedding planes. Glauconite content is 15% to 30%.	Low- to moderate-energy. Storm wave action. Deposition just below the fair-weather wave base. Frequent alternation of suspension fallout deposition interrupted by combined-flow deposition during storm events.	BI = 2-5<br><br>*Teichichnus rectus*<br>*Planolites* isp.<br>*Palaeophycus* isp.<br>*Cylindrichnus concentricus*<br>*Bergaueria* isp.	Transgressive deposits: distal bay	Earlie Formation
Sgl-Bio	**Bioturbated glauconitic sandstone**<br>Facies interval: 0.1-0.3 m thick. Dark green, very thin- to medium-bedded (2-30 cm thick), massive, moderately-sorted, glauconitic, fine-grained sandstone. Glauconite content is 35 to 50%. Few linguliform brachiopod shell fragments. Bioturbation intensity is moderate to high.	Low- to moderate-energy. Deposition just below the fair-weather wave base.	BI = 3-4<br><br>*Teichichnus rectus*<br>*Palaeophycus* isp.<br>*Planolites* isp.	Transgressive deposits: proximal bay	Earlie Formation
Sgl-Lst	**Interbedded glauconitic sandstone and dolostone**<br>Facies interval: 2-2.4 m thick. Interbedding of greenish gray, 1-4 cm thick, fine- to medium-grained, carbonate cemented, glauconitic (25-35%), fine-grained sandstone and light gray, 1 to 5 cm thick, glauconitic (1-10%) dolostone with small linguliform brachiopod shell fragments.	Low- to moderate-energy. Deposition between fair-weather wave base and storm wave base.	BI = 2-3<br><br>*Teichichnus rectus*	Transgressive deposits: distal bay	Earlie Formation
M	**Mudstone with discontinuous limestone/dolostone**<br>Facies interval: 3-15 m thick. Dark gray to green, very thin- to medium-bedded (1-30 cm thick), parallel- to cross-laminated mudstone with discontinuous light gray, very thin-bedded (1 cm thick) limestone/dolostone. Linguliform brachiopod shell fragments, hyoliths and trilobites.	Low energy. Suspension fallout deposition below storm wave base.	BI = 0-1<br><br>*Planolites* isp.<br>*Helminthopsis* isp.	Distal bay to shelf	Earlie Formation
M-Lst	**Interbedded mudstone and limestone/dolostone**<br>Facies interval: 0.5-13 m thick. Intercalated dark gray, very thin- to medium-bedded (1-20 cm thick), fissile, parallel-laminated, micaceous, slightly calcareous, mudstone with gray to white, very thin- to thin-bedded (1-5 cm thick) limestone and dolostone, and discontinuous layers of green, very thin-bedded (1-2 cm thick), parallel-laminated micaceous siltstone. Local calcareous nodules, clasts and lenses. Disseminated pyrite. Linguliform brachiopod shell fragments (calcium phosphate) and trilobites.	Low energy. Suspension fallout of fine clastics alternated with carbonate sediment precipitation.	BI = 0-2<br><br>*Planolites* isp.<br>*Helminthopsis* isp.	Distal bay to shelf	Earlie Formation

Lst	**Limestone/dolostone with discontinuous shale**<br>Facies interval: 0.3-0.5 m thick. Gray, pink and tan, very-thin to thin-bedded (1-9 cm thick), argillaceous, nodular and fossiliferous limestone with discontinuous dark gray, very thin-bedded (1-2 cm thick), parallel-laminated shale. Common linguliform brachiopod shell fragments.	Precipitation and accumulation of carbonate sediment in a low energy marine setting. Below the storm wave base.	BI = 0-2<br><br>*Planolites* isp.	Distal bay to shelf	Earlie Formation

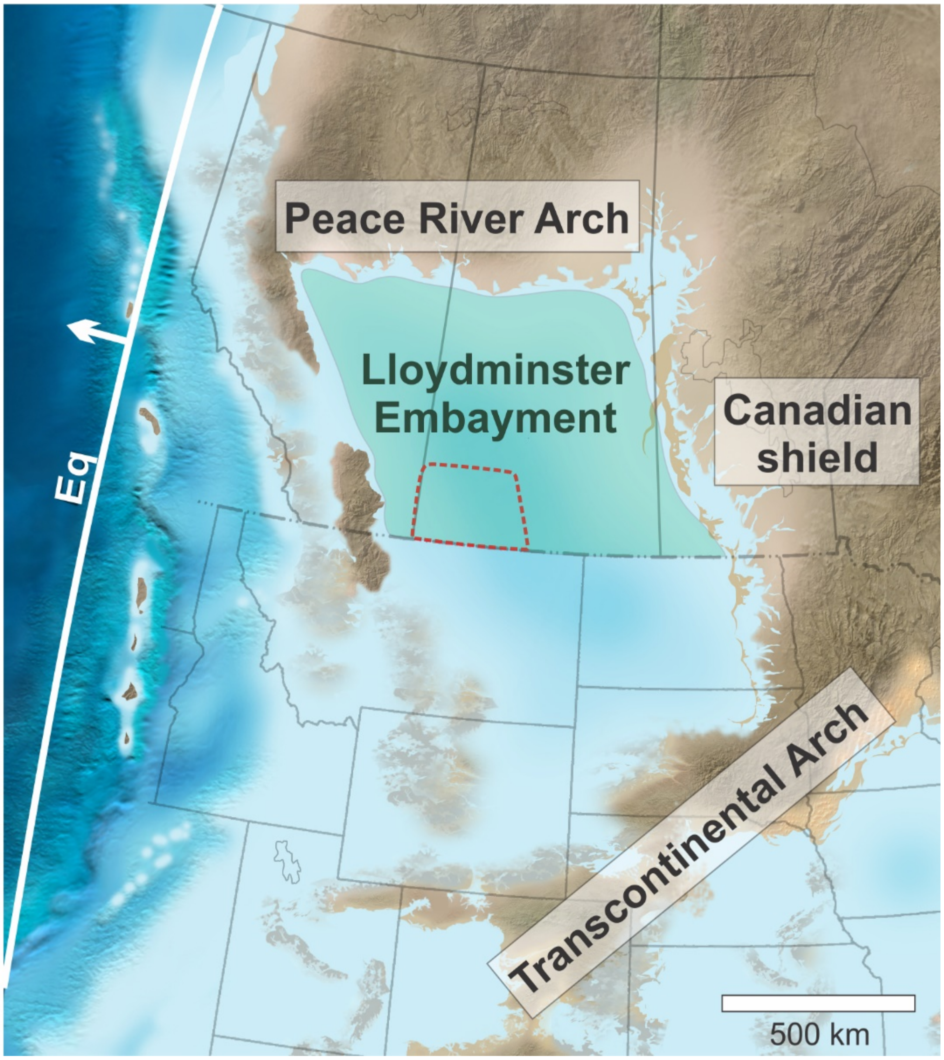

Figure S1. Paleogeographic map of the late Cambrian (ca. 500 Ma) centered on Laurentia. Source map © 2020 Colorado Plateau Geosystems Inc. Modified by A.I. using CorelDRAW 2017 v.19.1.0.419 software.

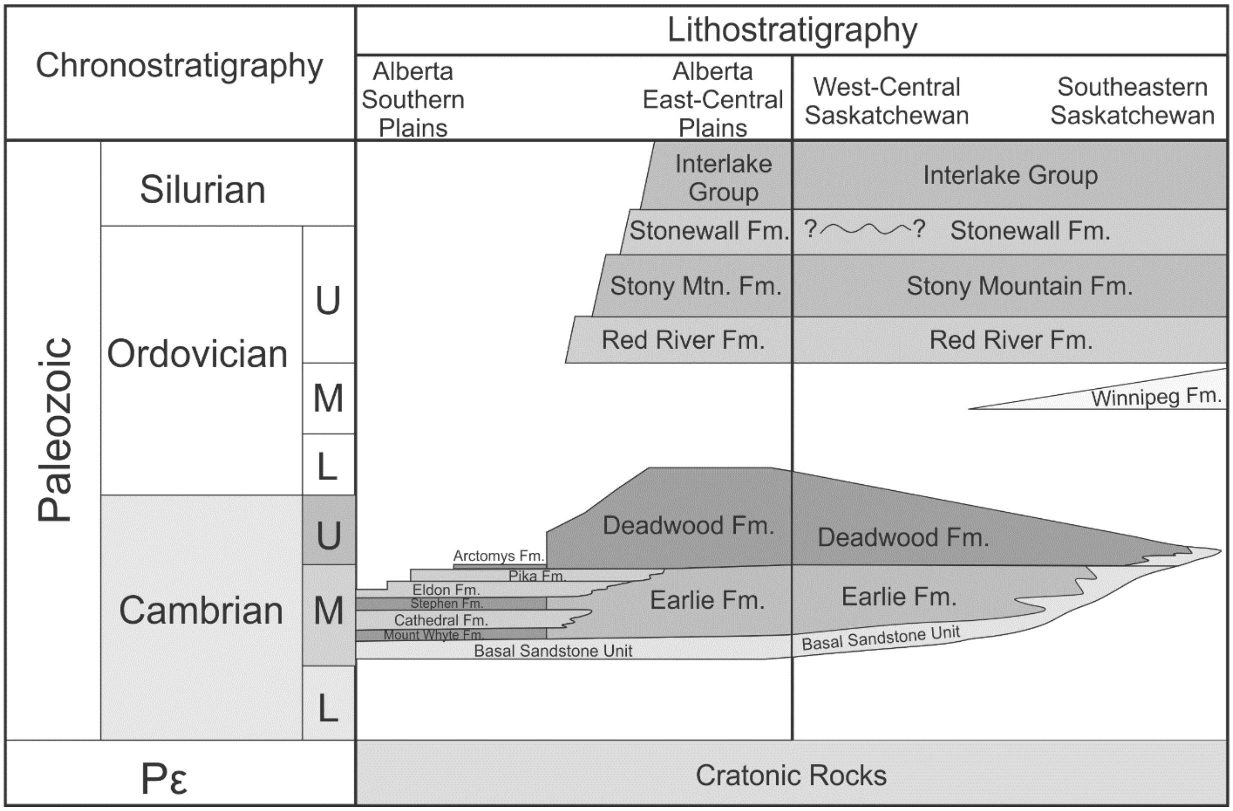

Figure S2. Cambrian-Silurian stratigraphy in Alberta and Saskatchewan. Figure drawn by A.I. in CorelDRAW 2017 v.19.1.0.419 software, based on stratigraphic chart found in [72].

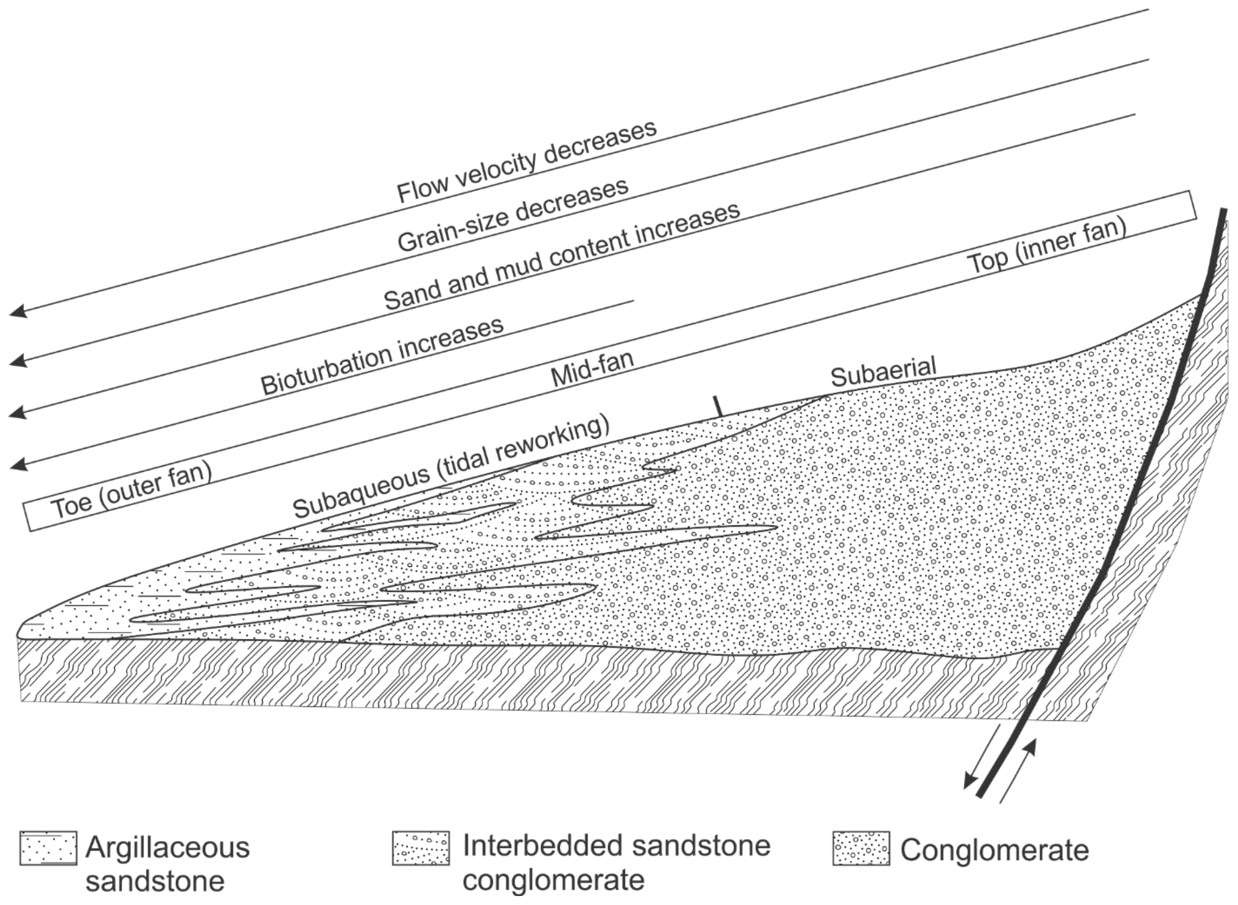

Figure S3. Architecture of gravel-rich delta-fan wedges, based on representation found in [73]. Drawn by A.I. using CorelDRAW 2017 v.19.1.0.419 software.

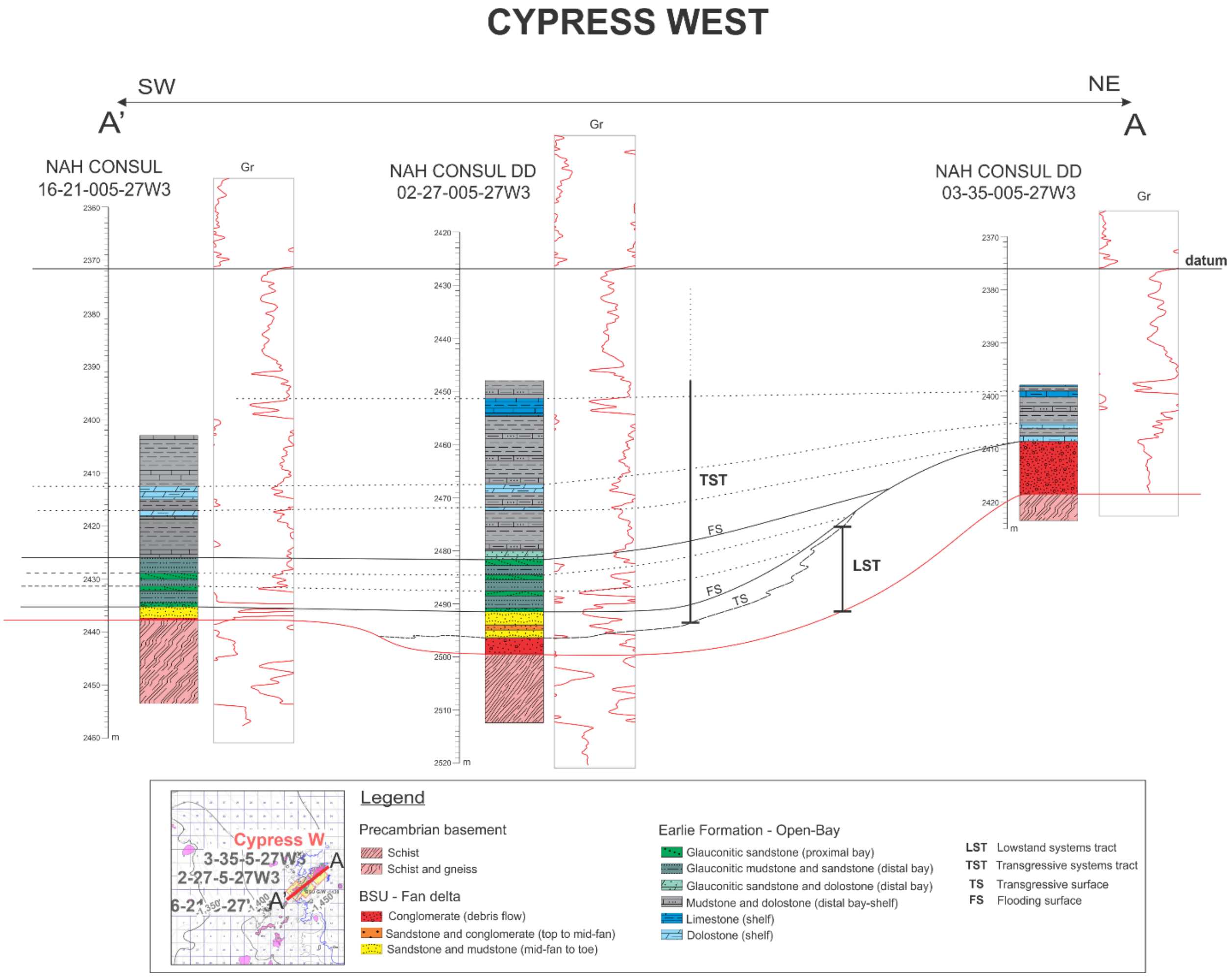

Figure S4. SW-NE stratigraphic correlation panel in the Cypress West area showing lateral distribution of the sedimentary facies and its sequence stratigraphic interpretation. Drawn by A.I. using CorelDRAW 2017 v.19.1.0.419 software.

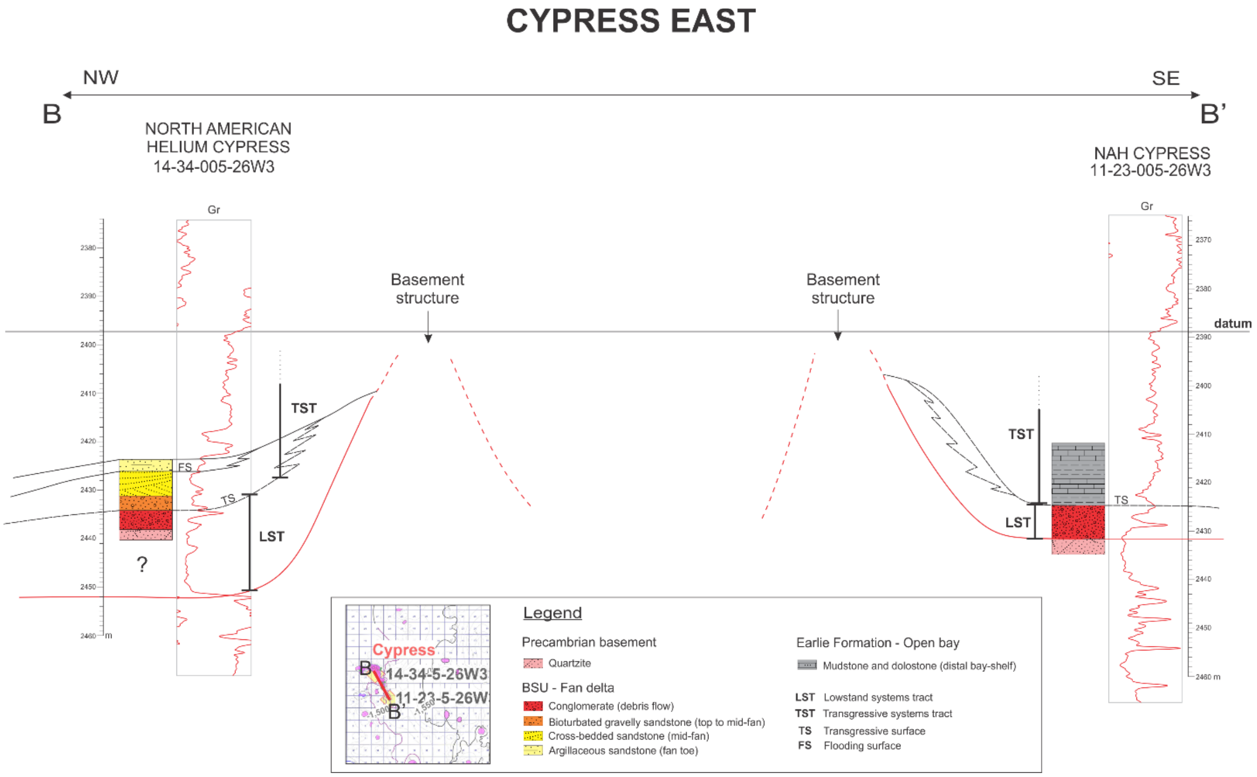

Figure S5. NW-SE stratigraphic correlation panel showing vertical successions at the outer flanks of two basement structures in the Cypress East area. Facies correlation between the structures is difficult due to lack of geophysical and well-core data. Drawn by A.I. using CorelDRAW 2017 v.19.1.0.419 software.

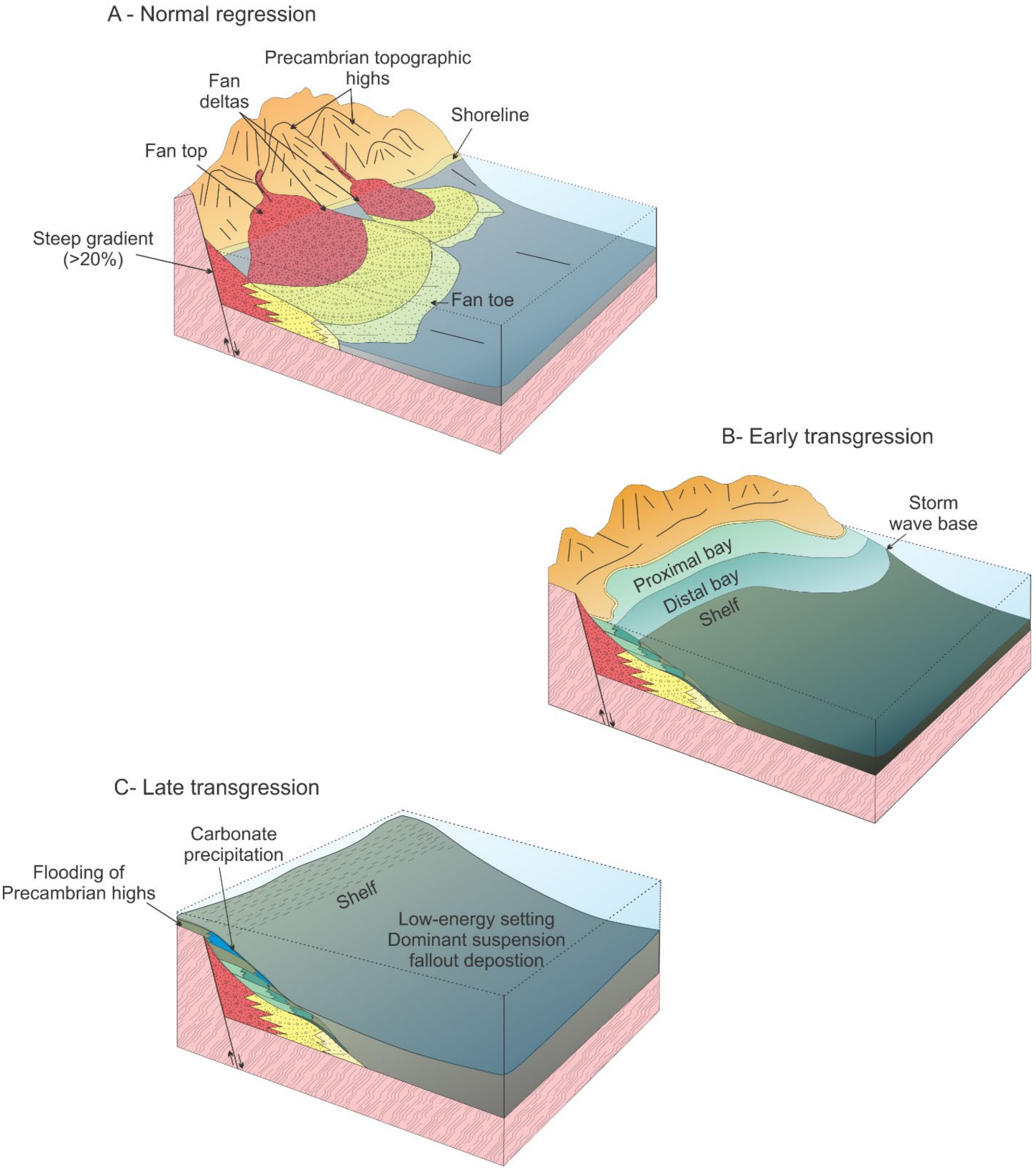

Figure S6. Paleoenvironmental evolution of successions close to uplifted and active Precambrian blocks. Models drawn by A.I. using CorelDRAW 2017 v.19.1.0.419 software.

References

72 Dixon, J. Stratigraphy and facies of Cambrian to Lower Ordovician strata in Saskatchewan. *Bull. Can. Pet. Geol*. **56**, 93-117 (2008).

73 Reading, H. G. & Richards, M. Turbidite systems in deep-water basin margins classified by grain size and feeder system. *AAPG Bull.* **78**, 792-822 (1994).
